# Genome-Wide Profile of Pleural Mesothelioma versus Parietal and Visceral Pleura: The Emerging Gene Portrait of the Mesothelioma Phenotype

**DOI:** 10.1371/journal.pone.0006554

**Published:** 2009-08-07

**Authors:** Oluf Dimitri Røe, Endre Anderssen, Eli Helge, Caroline Hild Pettersen, Karina Standahl Olsen, Helmut Sandeck, Rune Haaverstad, Steinar Lundgren, Erik Larsson

**Affiliations:** 1 Department of Oncology, St. Olavs Hospital, University Hospital of Trondheim, Trondheim, Norway; 2 Institute of Cancer Research and Molecular Medicine (IKM), Norwegian University of Science and Technology (NTNU), Trondheim, Norway; 3 Department of Laboratory Medicine, Children's and Women's Health (LBK), Norwegian University of Science and Technology (NTNU), Trondheim, Norway; 4 Institute of Community Medicine, University of Tromsø, Tromsø, Norway; 5 Department of Pathology and Medical Genetics, St. Olavs Hospital, University Hospital of Trondheim, Trondheim, Norway; 6 Department of Cardiothoracic Surgery, Bergen University Hospital, Bergen, Norway; Uppsala University, Sweden

## Abstract

**Background:**

Malignant pleural mesothelioma is considered an almost incurable tumour with increasing incidence worldwide. It usually develops in the parietal pleura, from mesothelial lining or submesothelial cells, subsequently invading the visceral pleura. Chromosomal and genomic aberrations of mesothelioma are diverse and heterogenous. Genome-wide profiling of mesothelioma versus parietal and visceral normal pleural tissue could thus reveal novel genes and pathways explaining its aggressive phenotype.

**Methodology and Principal Findings:**

Well-characterised tissue from five mesothelioma patients and normal parietal and visceral pleural samples from six non-cancer patients were profiled by Affymetrix oligoarray of 38 500 genes. The lists of differentially expressed genes tested for overrepresentation in KEGG PATHWAYS (Kyoto Encyclopedia of Genes and Genomes) and GO (gene ontology) terms revealed large differences of expression between visceral and parietal pleura, and both tissues differed from mesothelioma. Cell growth and intrinsic resistance in tumour versus parietal pleura was reflected in highly overexpressed cell cycle, mitosis, replication, DNA repair and anti-apoptosis genes. Several genes of the “salvage pathway” that recycle nucleobases were overexpressed, among them TYMS, encoding thymidylate synthase, the main target of the antifolate drug pemetrexed that is active in mesothelioma. Circadian rhythm genes were expressed in favour of tumour growth. The local invasive, non-metastatic phenotype of mesothelioma, could partly be due to overexpression of the known metastasis suppressors NME1 and NME2. Down-regulation of several tumour suppressor genes could contribute to mesothelioma progression. Genes involved in cell communication were down-regulated, indicating that mesothelioma may shield itself from the immune system. Similarly, in non-cancer parietal versus visceral pleura signal transduction, soluble transporter and adhesion genes were down-regulated. This could represent a genetical platform of the parietal pleura propensity to develop mesothelioma.

**Conclusions:**

Genome-wide microarray approach using complex human tissue samples revealed novel expression patterns, reflecting some important features of mesothelioma biology that should be further explored.

## Introduction

**Figure 1:**
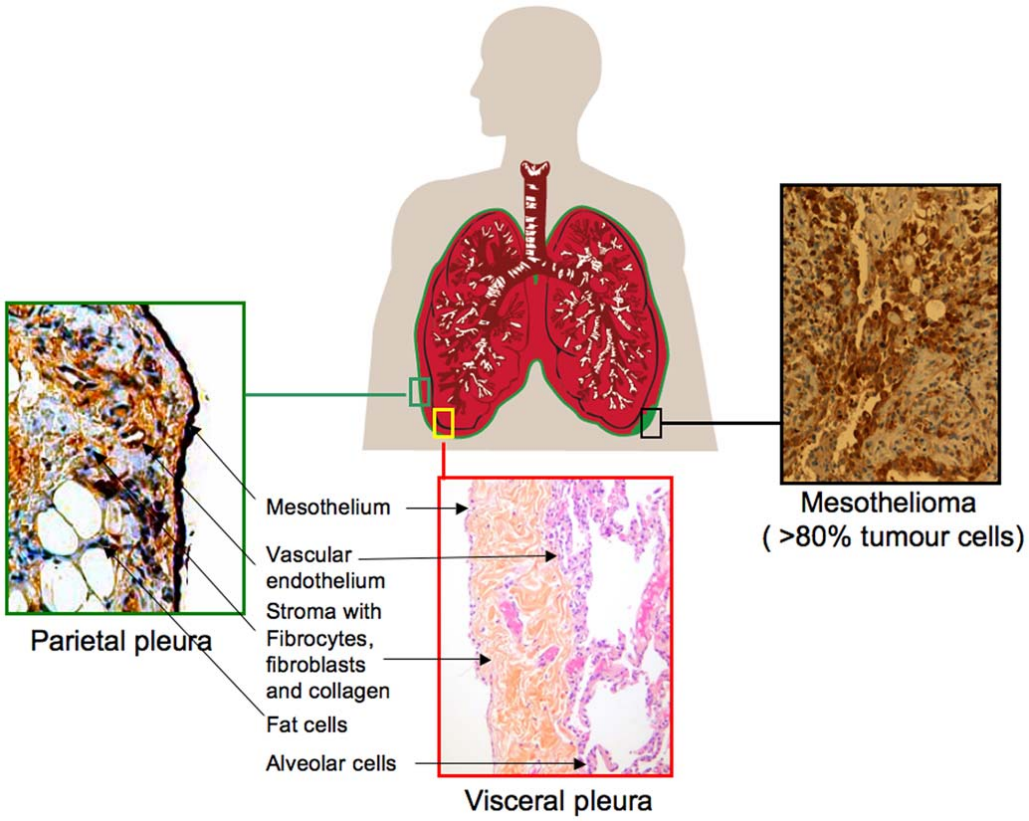
Schematic presentation of mesothelioma, the parietal and visceral pleura. Representative histology showing the most abundant cell types.

Malignant mesothelioma is an aggressive and incurable tumour with currently a median survival of 12 months[1]. Its inherent chemo- and radio-resistance has spread treatment nihilism over four decades[2]. Occasionally however, good responders and long-term survivors are seen. Mesothelioma is derived from cells of the pleura, peritoneum or tunica vaginalis, of which pleural location accounts for about 70% of the cases[3]. Epithelial subtype is the most common, and is an important positive prognostic factor in contrast to the sarcomatous and mixed subtypes. Mesothelioma predilection site is the parietal pleura (Fig. 1) where tumour grows in a loco-regional pattern, spreading to the visceral pleura and invade the surrounding structures[4]. Asbestos is the most important carcinogenic factor, but radiation can induce it and Simian virus 40 (SV40) has been implicated, but mainly as a co-factor[1]. Asbestos fibres are found both in the parietal and visceral pleura as well as in the lung. Why the parietal pleura and not the visceral pleura is the main target organ of mesothelioma is unknown, so a higher grade of susceptibility to oncogenic factors than the visceral pleura could be hypothesized.

Moreover cytogenetic studies have shown that mesotheliomas have highly complex and variable chromosomal aberrations[5], and only few common important features have been identified, as the deletion of 9p21 including the CDKN2A gene[6]. Consequently genome-wide microarray analysis may be a more fruitful method to identify the most important common and crucial genes and pathways involved in its biology. Genome-wide studies of pleural mesothelioma versus normal non-cancer parietal and visceral pleura have yet to be published. The main aim of this study was to analyze the gene profile of human pleural mesothelioma versus normal parietal and visceral pleural tissues, focusing on pathway analysis and differential gene expression correlated to gene function.

## Results

### Characterization of the patients and tissues

Gene expression analysis of six mesothelioma samples where two were from the same patient, seven parietal pleural samples where two were from the same patient and three visceral pleural samples were accomplished (Table 1). Mean age of controls was 27 years and of cases 56 years. None of the controls were reportedly ever exposed to asbestos, whereas four of five cases had various levels of exposure. Parietal pleura samples from the controls had normal histology, except case 2 that had partly reactive fibrosis (Table 2). The visceral pleural samples, that were from the same control patients were part of, or close to a bullae, described as bullous emphysema by histological examination, but none of the patients had an ephysema diagnosis nor clinical emphysema. By light microscopy of Hematoxylin-Eosin-Safranin-staining of normal tissue and diagnostic immunohistochemistry of the tumour samples we identified 17 cell-types (not shown), where four cell types mainly distinguished tumour from normal pleura. These were mesothelioma cells that were in abundance in the tumour samples, normal mesothelial, endothelial cells and fibrocytes in the normal pleura (Table 3). Larger vessels were more frequent in the parietal samples than the visceral. The visceral vessels were surrounded by leuko- and histiocytes, and in two of the visceral samples 30% of the cells were alveolar. Collagen was abundant in both visceral and parietal pleura.

**Table 1:** Description of cases (T) and controls (C).

ID	Cases	Age	Gender	Survival	History	Primary stage	Asbestos exposure years (y)	Smoking years (y)
1	C	25	M		Recurrent right-sided pneumothorax, apical and lateral right superior lobe bullae.	T0N0M0	0	4
2	C	16	F		Recurrent left-sided pneumothorax, apical bullae.	T0N0M0	0	0
3	C	27	M		Recurrent right-sided pneumothorax, apical bullae.	T0N0M0	0	12
4	C	51	M		Recurrent right-sided pneumothorax, multiple cysts superior lobe.	T0N0M0	0	34
5	C	19	M		Recurrent right-sided pneumothorax, apical bullae	T0N0M0	0	1
6	C	18	M		Left- then right-sided pneumothorax, apical bullae.	T0N0M0	0	0
7	T	58	M	15	Thoracic pain 6 months, then dyspnoea and expectorate, 6xCCG with partial remission, progression after 4xPC.	T2N2M1	Unsure, possible ecological	30
8	T	42	F	69	Dyspnoea 8 months, tumor in mediastinum, 6xCCG with partial remission, now 36xPC with excellent partial remission.	T4N3M0	Hair-dryer with asbestos elements, 9	6
9	T	71	M	11	Pain right thorax and dyspnoea 4 months, 5 kg weight loss, 2xpegylated doxorubicin, progression, 4xPC with stable disease	T2N2M0	Minimal	Not answered
10	T	50	F	6	Large tumour of right thorax involving the breast and mediastinum, radiotherapy 3Gy x 13 because of vena cava superior syndrome, no effect, new biopsy 1 month later, 1xCCG with haematological toxicity grade IV. No more treatment indicated.	T4N3M1	Unsure, worked in canning industry, old building	35
11	T	64	M	17	15 months breathless, weight loss 20 kg, blood-tinged pleural fluid, no pathological cells in pleural fluid after 3 months, tumour left pleura. 3xPC with progression, 6xCCG with clinical effect.	T2N1MX	40	35

PC = pemetrexed and carboplatin, CCG = pegylated doxorubicin, carboplatin and gemcitabine.

Survival was calculated in months from diagnosis (m).

**Table 2:** RNA isolation and histopathology.

ID	RNA	Histology P:positive, N:negative
1	PP two samples	Visceral pleura: Bullous emphysema*. Parietal pleura: Normal
2	PP and PV	Visceral pleura: Bullous emphysema. Parietal pleura: Reactive fibrosis and normal
3	PP	Visceral pleura: Bullous emphysema. Parietal pleura: ND
4	PP and PV	Visceral pleura: Bullous emphysema fibrous thickening. Parietal pleura: Normal
5	PP and PV	Visceral pleura: Bullous emphysema. Parietal pleura: Normal
6	PP	Visceral pleura: Emphysematous bullae. Parietal pleura: Normal
7	T	Epithelial type. P: Calretinin, EMA some positive cells, CK5/6. N: CEA, BerEp4, PSA,
8	T	Epithelial type. P: Calretinin, EMA moderate, CK 7. N: CEA, BerEp4, S-100, Chromogranin, Thyreoglobulin, Calcitonin, TTF-1, Synaptophysine, CK20
9	T	Epithelial type P: Calretinin, EMA, Pancytokeratin, CK5/6, Vimentin,MIB1 30%. N: CEA, BerEp4
10	T from two locations	Epithelial type, grade 3. P: Calretinin, EMA, BerEp4 (focal), Pancytokeratin, N: CEA, CK20, Estrogen, Progesterone, Erbb2
11	T	Biphasic type P: Calretinin- small groups, EMA, CK7 focal, CK5/6 some positive cells, Vimentin, BerEp4-focal. N: CEA, CK20, TTF-1, PSA, PSF

RNA was isolated from parietal pleura (PP) visceral pleura (PV) and mesothelioma (T).

* Controls were operated for spontaneous pneumothorax. Histology of the bullae that induced the pneumothorax showed that none had clinical or radiological emphysema.

**Table 3:** Cell types fraction of nucleated cells in parietal pleura (PP) and tumour (T).

% of nucleated cells in each biopsy							
ID		Epith. Comp mesothelioma	Sarc. Comp Mesothelioma	Mesothelium	Endothelium	Fibrocytes	Lymphocytes
1	PP	0	0	10	40	40	2
2	PP	0	0	1	30	50	2
3	PP	ND	ND	ND	ND	ND	ND
4	PP	0	0	1	20	50	1
5	PP	0	0	5	20	15	24
6	PP	0	0	15	40	20	10
7	T	55	0	<1	2	4	25
8	T	90	0	0	1	5	2
9	T	90	0	0	3	1	2
10	T	90	0	0	<1	3	2
11	T	25	15	<1	5	10	15

One of the two samples of case no. 10 is removed as non-representative histologically (see text). ND = not done.

### General expression characteristics

**Figure 2:**
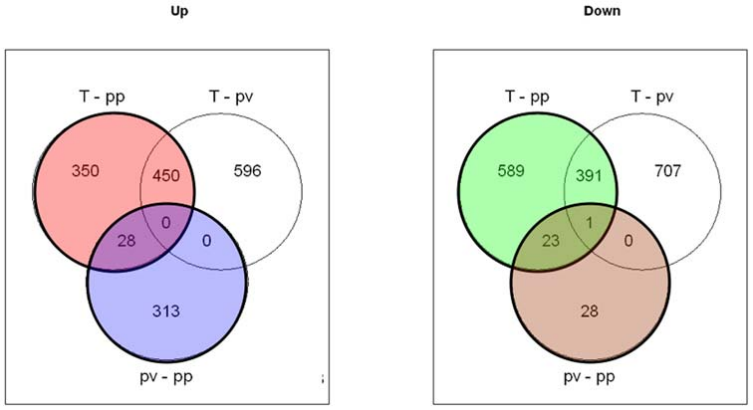
Venn diagram of significantly up- and down-regulated genes (n) in mesothelioma (T) versus normal parietal pleura (pp) and normal visceral pleura (pv) (*P*<0.05). 828 genes are overexpressed (red) and 1004 genes are down-regulated (green) in T versus pp. 341 genes are overexpressed (blue) and 52 genes downregulated (brown) in pv versus pp.

**Figure 3:**
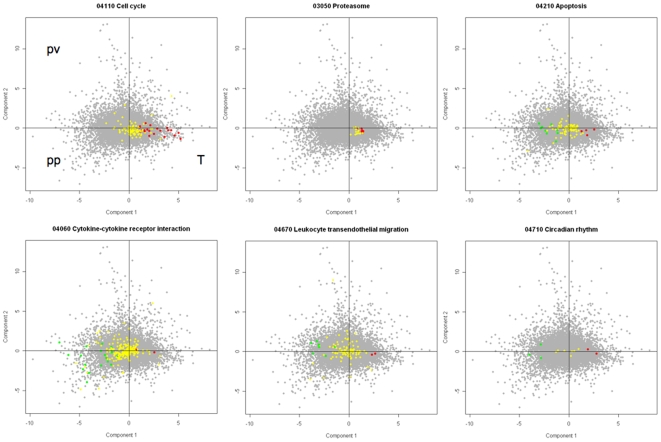
Selected pathways with distribution of differentially expressed genes (*P*<0.05). This graph depicts the areas of differentially expressed genes in tumour (T), parietal pleura (PP) and visceral pleura (PV). Each dot represents a gene, where red represent genes overexpressed in tumour and green represent genes overexpressed in parietal pleura or visceral pleura. Gray represents all the genes of the chip and yellow represents the genes non-differentially expressed in each pathway. Genes associated to the cell cycle and the proteasome are uniformly overexpressed. More genes associated to apoptosis are downregulated than overexpressed and most genes involved in cytokine-cytokine receptor interaction are down-regulated. Important circadian rhythm genes are differentially regulated (see Fig. 8).

PCA (principal component analysis) and a PLS (bridge-partial least squares regression) model showed that mesothelioma, parietal and visceral pleural tissues had distinct differential gene expression profiles[7]. Importantly there was higher inter-individual than intra-individual gene expression similarity between parietal and visceral pleura and there were more down-regulated than overexpressed genes in mesothelioma versus normal tissues ([7] and Fig. 2). KEGG PATHWAY analysis comparing the distribution of the gene expression of each pathway visualised in a graphic model, showed among others that the purine and pyrimidine metabolic pathways (not shown), cell cycle and proteasome, were selectively overexpressed in tumour (Fig. 3). Cytokine-cytokine receptor interaction, leukocyte transendothelial migration and apoptosis were mainly down-regulated in tumour (Fig. 3).

### Parietal versus visceral pleura

There were 392 differentially expressed genes between the normal parietal and visceral pleura, where 341 genes were down-regulated and only 52 genes overexpressed in parietal pleura (Fig. 2).

No gene ontology (GO) entities were overexpressed in parietal pleura but several entities were down-regulated compared to visceral pleura (Table 4). Among the most important were the genes intrinsic to membrane, signal transduction and adhesion genes. Single genes reflecting this was down-regulation of integrins (ITGA2, ITGB3, ITGA8), claudins (CLDN4, CLDN7), protein kinases (PRKCE, PRKCZ) and syndecan 1 (SDC1). In KEGG PATHWAYS focal adhesion and leukocyte endothelial migration was also down-regulated.

**Table 4:** A selection of down-regulated gene ontology (GO) entities and genes (Down Genes) in normal parietal pleura versus visceral pleural tissue.

GO terms	Down Genes	Genes on Chip	Corrected P-values
GO:0031224 intrinsic to membrane	105	4176	1,18E-05
GO:0050828 regulation of liquid surface tension	4	5	0,0014
GO:0007275 multicellular organismal development	51	1984	0,0033
GO:0004871 signal transducer activity	43	1797	0,0344
GO:0007155 cell adhesion	21	664	0,0425
GO:0045893 positive regulation of transcription, DNA-dependent	11	201	0,0287
GO:0006814 sodium ion transport	8	108	0,0317

Genes on Chip = the number of genes from each entity represented on the gene chip.

### Mesothelioma versus parietal pleura

Since the parietal pleura is the predilection site for mesothelioma, we further compared the mRNA expression of mesothelioma with normal parietal pleura. There were 828 overexpressed and 1004 down-regulated genes in tumour tissue (Fig. 2). Of these, 75 and 75 respectively, had no Unigene annotation, nor a gene symbol. GO entities involved in important biological functions including cell cycle, DNA repair and microtubule cytoskeleton genes were highly overexpressed (Table 5 and [7]).

**Table 5:** A selection of overexpressed gene ontology (GO) entities with corresponding genes (Genes Up) in mesothelioma versus parietal pleura.

GO term	Genes Up	Genes on Chip	Corrected P-value	Gene Symbols
GO:0007049 cell cycle	82	802	1,33E-15	
GO:0006260 DNA replication	27	181	3,51E-08	PHB,PCNA,TOP2A,RRM1,MCM3,MCM6,CDK2AP1,MCM2,TYMS,SSBP1,MSH6,RNASEH2A,RFC5,CDC6,RFC4,RBM14,FEN1,GINS1,GLI2,DNA2L,PRIM2,PTMSGTPBP, GMNN, ORC6L,GINS2,MCM4
GO:0000087 M phase of mitotic cell cycle	33	200	5,57E-11	RAD21,RAN,RUVBL1,SMC4,BIRC5,CCNB2,TXNL4A,CDC20,BUB1B,CDC6,ZWINTNDC80,SMC2,CDC25A,KIF23,CENPF,AURKA,BRCA2,KIF2C,BUB1,CDC2,NUDC,NCAPD3,NUSAP1,CEP55,ASPM,CDC23,RCC2,CCNB1,CLASP1,CIT,HELLS,ESPL1
GO:0006139 nucleobase, nucleoside, nucleotide and nucleic acid metabolic process	184	3337	1,62E-09	
GO:0005783 endoplasmic reticulum	61	731	1,01E-08	

Genes on Chip = the amount of genes from each entity represented on the gene chip. Due to lack of space not all overexpressed genes are shown under Gene Symbols. Some important genes and entities are discussed in the text.

Down-regulated GO entities were related to multicellular organism development and cell communication, defense, cell adhesion and interestingly, several circadian rhythm genes (Table 6). Moreover important tumor suppressor genes as DLC1 (deleted in liver cancer 1), TNF (tumour necrosis factor), CAV1 (caveolin-1) and GSN (gelsolin) were down-regulated. Contrary to other cancers, the well-known anti-apoptotic BCL2, the FOS oncogene and the multidrug resistance gene ABCB1 (ATP-binding cassette sub-family B member 1) were down-regulated.

**Table 6:** A selection of down-regulated gene ontology (GO) entities and corresponding genes (Genes Down) in mesothelioma versus parietal pleura.

GO term	GenesDown	Genes On Chip	Corrected P-value	Gene Symbols
GO:0007275 multicellular organismal development	129	1984	2,08E-05	
GO:0048511 rhythmic process	10	61	0,0254	HLF,NR1D1,EGR2,EGR3,STAT5BCRY2,ANG,PER3,TEF, PER1
GO:0030528 transcription regulator activity	93	1300	2,33E-05	
GO:0045934 negative regulation of nucleobase, nucleoside, nucleotide and nucleic acid metabolic process	28	290	0,0029	
GO:0007154 cell communication	214	3326	1,80E-08	
GO:0007165 signal transduction	197	3000	1,80E-08	
GO:0007264 small GTPase mediated signal transduction	34	418	0,0080	
GO:0007155 cell adhesion	59	664	1,45E-05	
GO:0006952 defense response	49	500	1,45E-05	
GO:0006954 inflammatory response	33	271	2,33E-05	
GO:0003707 steroid hormone receptor activity	10	49	0,0069	NR4A1,NR1D1,NR3C2,NR5A2, PGRMC2,NR4A2,NR1D2,PPARA, THRB,NR3C1

### Verification of protein expression

All samples of tissue adjacent to the tissue subjected to microarray, except control no. 3 where analysed by immunohistochemistry for protein expression of six selected genes. Due to limited biological material (needle biopsies) we had to be very selective in chosing which genes to verify. Overexpression was verified for Thymidylate Synthase, VG5Q, Chk1, NQO1 and RAD21, where tumour cells were positive in most cases. Normal mesothelial cells, that was a minor population of the biopsies (Table 3) stained positive for NQO1 and VG5Q, weakly for RAD21. MSLN (Mesothelin) mRNA was not differentially expressed, despite its strong protein expression in mesotheliomas[8]. Mesothelin protein was highly expressed in both mesothelial and stromal cells of the control samples, that could explain the non-differential expression of MSLN mRNA.

**Figure 4:**
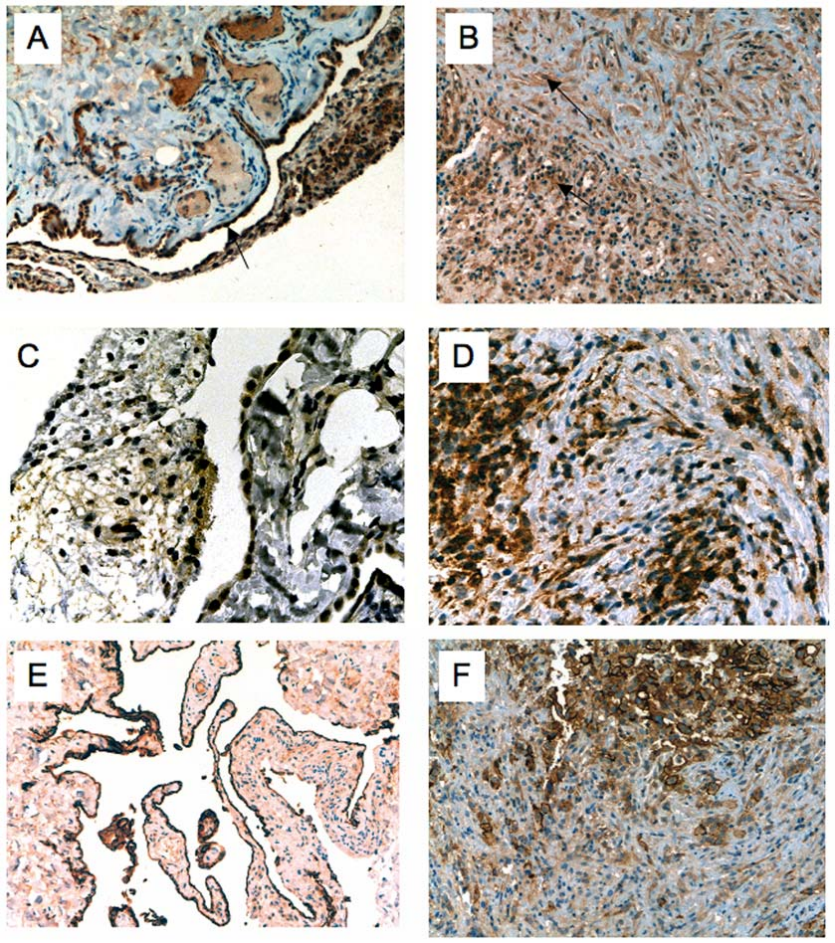
Protein expression of selected genes, AGGF1, TYMS and MSLN by immunohistochemistry. A–C–E: normal parietal pleura. B–D–F: Biphasic mesothelioma with epithelial and sarcomarous components. A–B (x20): AGGF1(VG5Q) mRNA was overexpressed in mesothelioma, and clearly protein was expressed (brown) in both tumour components (arrows). Strong expression in normal mesothelium was seen (arrow) but the majority of endothelial and other pleural cells were negative. C–D (x40): TYMS (Thymidylate synthase) mRNA was overexpressed, also on the protein level (brown), mostly in the epithelial component (arrow) of tumour. Normal pleura was negative. E–F (x20): MSLN (Mesothelin) mRNA was not differentially expressed, that could be explained by the intense protein expression not only in epithelial tumour cells, but also in normal mesothelial and stromal cells.

Histological pictures of normal parietal samples and biphasic mesothelioma stained with VG5Q, Thymidylate Synthase, and Mesothelin antibodies are shown illustrating the expression in normal pleura and the malignant epithelial and sarcomatous components (Fig. 4).

## Discussion

Genome-wide profiling of malignant pleural mesothelioma versus normal parietal pleura showed several new and interesting expression patterns highly relevant to the biology of mesothelioma. The gene expression differences between the parietal and visceral pleural tissues described here for the first time were significant and may be important for understanding the parietal pleura propensity for developing mesothelioma. Many of those features have been recognised mainly in epithelial malignant tumours, as will be discussed below, thus showing important genotypic similarities between this tumour of probably mesodermal origin and epithelial cancers. Moreover, 150 differentially expressed genes without known function were identified that may gain importance in the future.

When interpreting gene expression data one must also keep in mind that they represent relative values, so that overexpression e.g. in tumour also could reflect down-regulation in the normal tissue.

### Parietal versus visceral pleura

There were significant expression differences between these two pleural membranes. Interestingly the expression of the visceral pleura of case 2, 4 and 5 was much more alike than the parietal pleura of 2, 4 and 5 that formed a separate cluster, showing that tissues with a similar phenotype also share gene expression profile characteristics[7]. We do not claim that the mesothelial cells of these two membranes have different profiles, as these cells were not microdissected and analysed separately, but the sum of gene expression from all the cells give a picture of the activities of the two membranes. A large proportion of the differentially overexpressed genes of the visceral pleura, 105/341 genes (Table 4) were intrinsic to membrane, and the multiple functions of transporters and channels as well as genes with unknown functions. Several solute carrier family members (SLC) were down-regulated in the parietal pleura, transporters of multidrug and toxic compounds (SLC47A1), sodium-phosphate (SLC34A2), oligopeptide (SLC15A2), amino acid (SLC6A14), glutamate (SLC1A1), sodium/myo-inositol (SLC5A3) and glucose (SLC5A9) transporters. Interestingly the proton exchange transporter gene NHE1 (SLC9A1) that is important for tumour metastasis was down-regulated, as well as the sodium channel transporters SCN1A, SCNN1B and SCN7A. AQP4, aquaporin 4 was down-regulated as well, a gene important for water transport but also for cell migration and metastasis. Of the transporter genes, only the zink transporter (SLC30A1/ZNT1) was overexpressed in the parietal pleura, a gene that is overexpressed in the lung response to cobalt[9]. The presence of alveolar cells in the visceral pleural samples is clearly reflected as four of five genes encoding surfactant proteins were overexpressed in the visceral samples (Table 4). The microscopic emphysema seen in the visceral pleural samples could influence the gene profile, but this is unlikely as adhesion genes as claudins, integrins and laminins were highly overexpressed, reflecting the physiological phenotype of the visceral pleura (Table 4)[10]Among the few genes over-expressed in parietal pleura were PCOLCE and PCOLCE2, encoding procollagen proteinase enhancers, important in formation of normal collagen fibrils, and thus show that the expression represents collagen-rich pleural tissue[11]. Parietal pleura has lymphoid tissue (Kampmeier's foci) and is highly active in both production and transport of pleural fluid[10], but these were not detected histologically and not translated to gene expression. One explanation may be that these foci are predominantly found in the basal parts of pleura, and our samples were from more cranial areas. There are no obvious explanations why signal transducer activity, multicellular organismal development and leukocyte trans-endothelial migration genes are down-regulated in parietal pleura. These features were similar to what was found in mesothelioma versus parietal pleura. As an example, ITGA2 (integrin alpha 2) was downregulated in parietal pleura, a membrane adhesion protein which polymorphisms are associated to breast and prostate cancer[12], [13]. One could speculate if some of these expression patterns represent a transforming susceptibility profile of the parietal pleura. However, due to the abovementioned uncertainties, the small number of visceral samples and the fact that the parietal pleura is the principal site of mesothelioma, in all further comparisons with tumour the parietal pleura was used.

### Mesothelioma versus parietal pleura

Importantly there were more down-regulated than overexpressed genes in tumour versus parietal tissue corresponding with the recent findings of more chromosomal losses than gains in mesothelioma[5]. Analyzing the data within the KEGG PATHWAYS and GO revealed several important pathways and functions reflecting the aggressive and resistant phenotype of mesothelioma and some of the novel and most interesting findings will be highlighted below.

### Nucleotide metabolism

**Figure 5:**
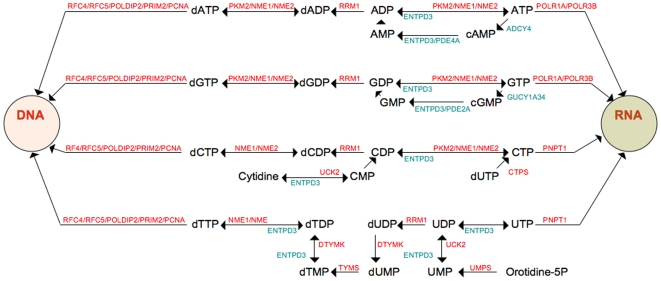
Schematic presentation of the results of differential expression of the purine and pyrimidine pathways in tumour versus parietal pleura (*P*<0.05). Genes encoding proteins responsible for DNA and RNA synthesis and recycling of purines and pyrimidines are overexpressed (red), while genes having the opposite or regulating role (green) are down-regulated. Genes encoding *de novo* synthesis of adenosine, guanosine, thymidine, cytidine and uracil were not differentially expressed (not shown). This pattern may represent salvage pathways facilitating tumour growth. Up: CTPS = CTP synthase, DTYMK = deoxythymidylate kinase, TYMS = thymidylate synthase, UCK2 = uridine-cytidine kinase, UMPS = uridine monophosphate synthase, POLR1A = polymerase (RNA) I polypeptide A, POLR3B; polymerase (RNA) III (DNA directed) polypeptide B, NME = non-metastatic cells 1, NME2 = non-metastatic cells 2, PKM2 = pyruvate kinase, muscle, PRIM2A = primase, DNA, polypeptide 2, PNPT1 = polyribonucleotide nucleotidyltransferase 1, PCNA = proliferating cell nuclear antigen, RRM1 = ribonucleotide reductase M1. Down: ADCY4 = adenylate cyclase 4, GUCY1A3 = guanylate cyclase 1, soluble, alpha 3, PDE2A = phosphodiesterase 2A, cGMP-stimulated, PDE4A = phosphodiesterase 4A, cAMP-specific, PDE5A = phosphodiesterase 5A, cGMP-specific, ENTPD3 = ectonucleoside triphosphate diphosphohydrolase 3.

As an expression of rapidly dividing cells, polymerases for RNA and DNA synthesis were overexpressed as well as genes of the purine and pyrimidine metabolism, but strikingly this was confined to genes of the so-called “salvage pathways”, where nucleobases are recycled rather than synthesized de novo[14], [15](Fig. 5). TYMS was overexpressed, encoding thymidylate synthase, part of the “salvage pathway” in mammals and known as the target of the antifolate drug pemetrexed that is active in mesothelioma. Its overexpression may confer to chemotherapy resistance and poor prognosis in other tumours, and recently TYMS has been regarded as an oncogene[16]. DTYMK (deoxythymidylate kinase), a key kinase for deoxythymidylate synthesis and involved in 5-Fu resistance was overexpressed[17]. A novel finding was PKM2 (pyruvate kinase muscle 2) overexpression. It is generally overexpressed in malignant tumours and encodes a key enzyme that regulate the ATP:ADP and GTP:GDP ratios in tumour cells and pooling of phosphometabolites that is a prerequisite for nucleotide biosynthesis. The tetrameric form of this protein is cleaved by oncoproteins such as the HPV16 E7[18] and the dimer is detected in serum and in the faeces of gastrointestinal cancer patients serving as a tumour marker[19]. Importantly NME1 and NME2 (non-metastatic cells 1 and 2), diphosphorylases that transfer phosphate groups between di- and trinucleotides (Fig. 5) were overexpressed. They are also associated to metastasis suppression in many cancer types[20]. Mesothelioma has mainly a non-metastatic growth pattern and overexpression of these genes may contribute to this phenotype.

### Genes involved in cell cycle function

**Figure 6:**
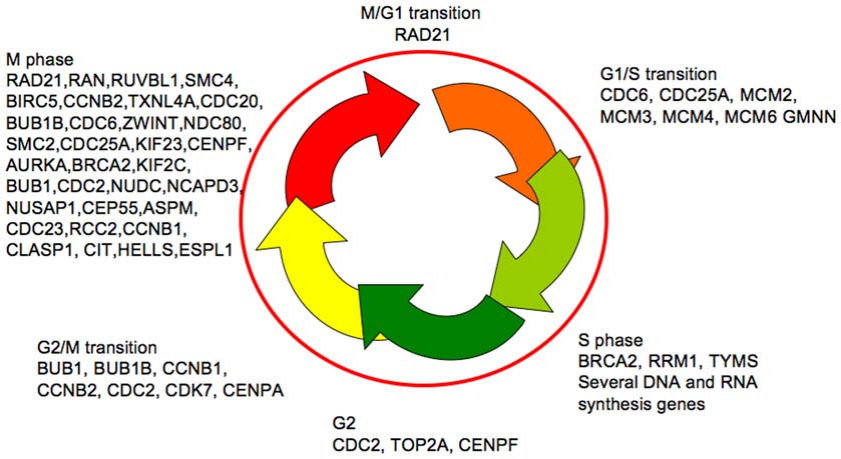
Schematic presentation of some of the overexpressed genes related to their activity in the various phases of the cell cycle (*P*<0.05). The M-phase genes are overrepresented.

**Figure 7:**
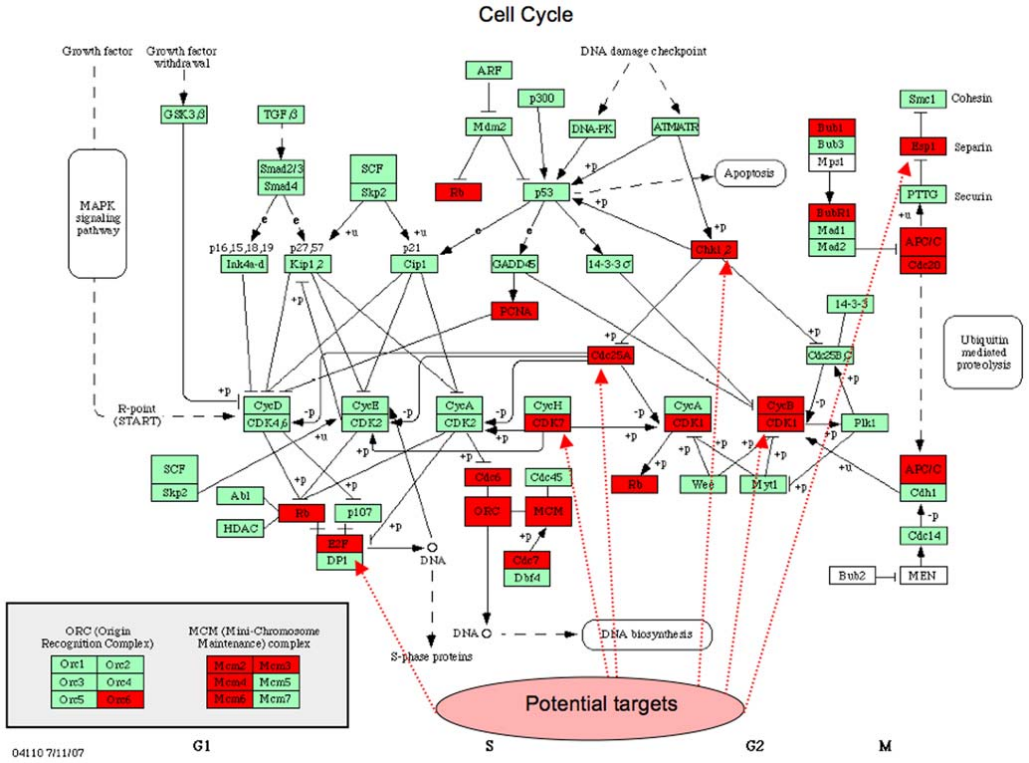
Differentially overexpressed genes in tumour (red boxes) depicted in the Cell Cycle map from KEGG PATHWAYS (Kanehisa *et al*., 2008) (*P*<0.05). 21 of 21 cell cycle genes were overexpressed in mesothelioma versus normal parietal pleura tissue. Potential targets for anti-tumour treatment described in the litterature are marked (see text). Abbreviations: CDK7 = cyclin-dependent kinase 7, CHEK1 = checkpoint homolog, E2F2 = E2F transcription factor 2, ORC6L = origin recognition complex, subunit 6 like, MCM2-3-4-6 = minichromosome maintenance complex component 2-3-4-6, PCNA = proliferating cell nuclear antigen, RB1 = retinoblastoma, BUB1 = budding uninhibited by benzimidazoles 1 homolog, BUB1B = BUB1 beta, CDC7 = cell division cycle 7 homolog, APC/C = CDC23, cell division cycle 23 homolog, anaphase-promoting complex subunit 8, CCNB1 = cyclin B1, CCNB2 = cyclin B2, ESPL1 = extra spindle pole bodies homolog 1, CDC2/CDK1 = cell division cycle 2, G1 to S and G2 to M, CDC6 = cell division cycle 6 homolog, CDC20 = cell division cycle 20 homolog, CDC25A = cell division cycle 25 homolog A.

It is known that cell cycle deregulation is a general feature of malignancy. Overexpression of the cell cycle, replication and M-phase genes reflect the importance of this also in mesothelioma (Fig. 6 and 7, Table 5). Genes driving all the phases of the cell cycle were significantly overexpressed (Fig. 6). No cyclins or cyclin dependent kinases (CDKs) that drive the cell cycle were down-regulated. Several of these genes are related to oncogenesis and/or have been proposed as anti-cancer targets for other tumours (Fig. 7) and some will be discussed here.

The overexpressed CDC6 encodes a protein essential for the initiation of DNA replication but has recently been shown to possess oncogenic properties by suppression of the INK4/ARF[21]. During the transition from a growth-arrested to a proliferative state transcription of mammalian Cdc6 is regulated by E2F proteins. E2F1-8 is a family of transcription factors with repressor or stimulator effect. E2F2 and E2F7 are overexpressed where the first is shown to be an activator and considered as an oncogene, overexpressed in large size and aggressive ovarian cancers[22]. The E2F transcription factors can be blocked by the tumour suppressor protein pRb encoded by RB1 that was overexpressed. In contrast to other cancers RB1 is rarely mutated in mesothelioma but its suppressor function is inhibited due to inactivation by phosphorylation or by viruses as SV40[23] that recently was linked to mesothelioma oncogenesis. CDKN2A (cyclin-dependent kinase inhibitor 2A) encoding the p16ink4a that inhibits pRb phosphorylation is almost always deleted in mesothelioma[6], resulting in normal but non-functional pRB expression, was not differentially expressed. We detected down-regulation its alternative reading frame gene, CDKN2AIP (CDKN2A interacting protein). CDKN2AIP activates the important tumour suppressor p53[24], consequently its down-regulation could as well be important for mesothelioma progression.

Essential for the initiation of eukaryotic genome replication are the MCM (mini-chromosome maintenance protein) complex that consist of MCM2-7, proteins possessing DNA helicase activity, and may act as a DNA unwinding enzymes. GMNN (geminin) regulate this complex and ensures genomic stability in cycling cells by preventing firing (or activation) of new replication origins before completion of a mitotic cycle, to ensure that DNA is replicated only once per cell cycle. MCM2, 3 and 6 that were overexpressed in our material (Table 5) are associated to poor prognosis in lung cancer[25], astrocytoma[26] and craniopharyngeal carcinoma[27] respectively. MCM3 is overexpressed in multiple malignancies, regarded a more sensitive tumour marker than Ki67, and 90% of mice injected with MCM3 transfected cells developed epithelial tumours within 6 weeks[28]. MCM4 combined with GMNN overexpression as found in our material, is also predictive for metastasis and poor survival in melanoma, documented in a large prospective microarray study[29]. Geminin may become a treatment target, as suppression by apigenin inhibited pancreatic cancer cell replication *in vitro* [30].

PRKCI (protein kinase C iota) is a serine- threonine kinase involved in cell cycle regulation by controlling the key cell cycle regulator CDK7[31] and both were overexpressed. PRKCI is also considered as an oncogene activated by nicotine and a critical gene in lung cancer development, conferring cell survival, drug resistance, migration and invasion[32], [33]. CDK7 encodes a protein that is required for assembly of the Cdk1(cdc2)/cyclin B1 complex and mitotic entry[34]. This protein is thought to serve as a direct link between the regulation of transcription and the cell cycle[35]. Inhibition of CDK7 by gambogic acid induced irreversible arrest of G2/M phase in gastric cancer cells, and is thus a putative treatment target[36]. CCNB1 encoding cyclin B1 and CDK1 encoding cdc2 were overexpressed, as in many cancer types, both essential components of the cell cycle regulatory machinery. Mesothelioma cells treated with alpha- interferon were blocked in the G2/M phase and cyclin B1/cdc2 expression was down-regulated[37]. Another gene encoding a protein essential for cell cycle progression through the G2/M transition, CDC23/APC subunit 8 was overepressed. This APC (anaphase-promoting complex) catalyzes the formation of cyclin B-ubiquitin conjugate that is responsible for the ubiquitin-mediated proteolysis of B-type cyclins and is also associated to tumorigenesis[38]. CDC20 is required to activate ubiquitin ligation by the APC and appears to act as a regulatory protein interacting with several points in the cell cycle, among them two microtubule-dependent processes, nuclear movement prior to anaphase and chromosome separation[39].

ORC6L is also overexpressed and is an essential gene that coordinates chromosome replication and segregation with cytokinesis and is overexpressed in colorectal cancer versus normal colon tissues[40]. ESPL1 (separase) is crucial in separating the sister chromatids at the moment of anaphase, and has also been proposed as a drug target in cancer[41].

Recently in a genome-wide study of localised melanomas that did or did not metastasize within four years, DNA replication genes were highly overexpressed in the metastatic group. In our material 10 genes out of their 35 were overexpressed (GMNN, CDC6, CENPF, MCM3, MCM6, ORC6L, PCNA, PTTG1, RFC4 and RFC5) and several other negative prognostic genes were common with our study (BIRC5/survivin, BUB1, CCNB1, CDC2, CENPA and MCM4)[29], [42], rendering the replicative system very important for future target development.

### Circadian rhythms

**Figure 8:**
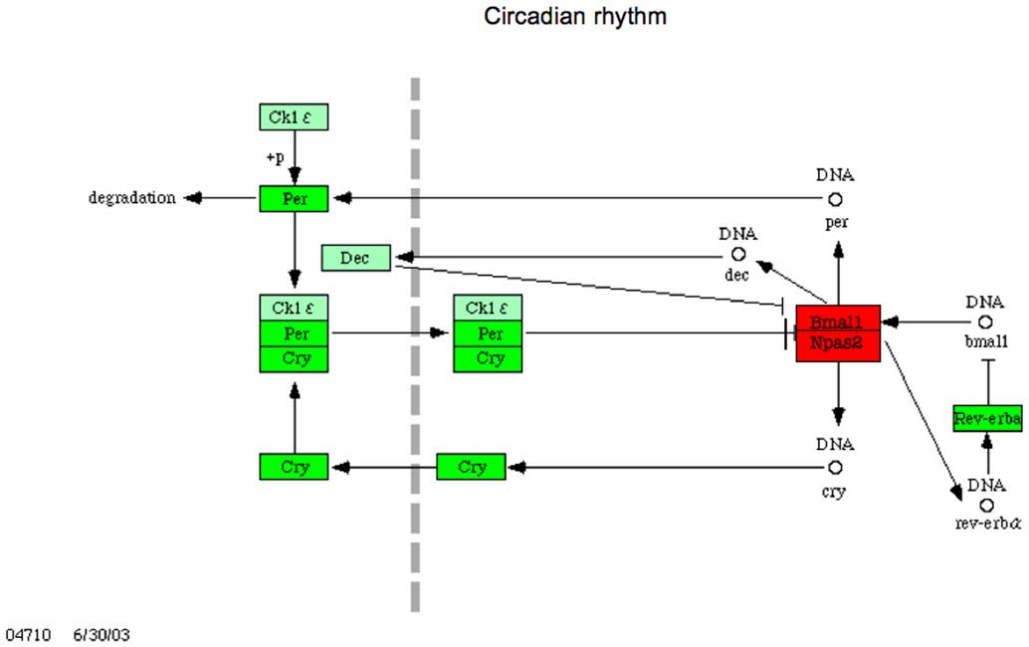
Circadian rhythm genes differentially expressed in tumour shown with KEGG PATHWAYS (modified from Kanehisa *et al*., 2008) (*P*<0.05). CRY2, PER1, PER3 and NR1D1/Rev-Erb alpha that function as negative regulators of transcription are down-regulated (green) whereas both genes encoding the active transcriptional heterodimeric complex Bmal1(ARNTL):Npas2 (NPAS2) are overexpressed in mesothelioma versus normal parietal pleura. Damaged circadian rhythms may be a key to the continuous replicative force in tumour cells, and thus possible treatment targets.

Circadian rhythm genes have recently been related to replication, damage responses and carcinogenesis and may play a master role in cell division[43]. We found central circadian clock genes differentially expressed (Table 6 and Fig. 8). The negative regulators of the cell cycle PER (period) and CRY (cryptochrome) genes, and their protein expression are downregulated in breast and lung cancer tissue when compared with matched normal tissue, as was found here, and methylation rather than mutation of these genes confer to this phenotype [44], [45], [46]. Cellular experiments have shown that their down-regulation confer resistance against apoptosis. NR1D1 and NR1D2 encode RevErb alpha and RevErb beta, two other negative regulators of the mammalian clock and repressors of transcription were downregulated as well, and their role in cancer is currently investigated[47]

Moreover, we found the positive regulators of circadian rhythms and cell cycle ARNTL/BMAL1 (aryl hydrocarbon receptor nuclear translocator-like) and its heterodimer NPAS2 (neuronal PAS domain protein 2)[48] overexpressed. Importantly, circadian BMAL1 expression was in tumour of a mouse model followed by TYMS expression and combined overexpression correlated to low response and worse survival on 5-Fu treatment[49]. We also found concomitant BMAL1 and TYMS overexpression indicating that this clock gene may also be an important driver of mesothelioma progression. Conversely, BMAL1 knockout conferred cyclohosphamide sensitivity and CRY knockout conferred cyclophosphamide resistance, showing that circadian genes are important in drug resistance as well[50]. High mRNA levels in breast cancer of the positive regulator TIMELESS has been significantly associated with shorter relapse-free survival and recently been regarded as a promising marker of tamoxifen resistance in women with estrogen receptor alpha-positive breast tumors[51]. TIMELESS was also overexpressed in the mesothelioma samples. The significant overexpression of positive clock genes with concomitant down-regulation of their negative counterparts seen here may be one of the basic regulator mechanisms of mesothelioma cell division, and thus in theory be an important pathway to target.

### Apoptosis

Apoptotic pathways and genes therein were mainly down-regulated in contrast to anti-apoptotic genes which were overexpressed (Fig. 3). Genes encoding proteins activating the anti-apoptotic NFkB (nuclear factor kappa beta) pathway were overexpressed, among them IL1RAP (interleukin 1 related accessory protein)[52] and PRKCA (protein kinase C alpha). PRKCA is also overexpressed in glioma and small-cell lung cancer and involved in several pathways of signal transduction, cellular communication and immune system, among them the VEGF and the ErbB signalling pathway[53].

AURKA (Aurora kinase A) was overexpressed, and in mammalian cells overexpression leads to centrosome amplification, genetic instability and transformation, as well as cisplatin resistance. Its activation of the NFkB pathway has been proposed as an important mechanism[54]. AURKA is overexpressed in several cancers, and has been associated with shorter survival in mesotheliomas[55]. Small molecule inhibitors of AURKA are currently in phase II trials[56]. The important inhibitor of apoptosis BIRC5/survivin that confers drug resistance and tumour aggressiveness was also overexpressed, and discussed in [7].

### Angiogenesis

Angiogenesis is important for tumour progression and survival [57], and antiangiogenic therapies targeting the VEGF and VEGFR have been developed. VEGF protein is highly expressed in mesothelioma [58], but the mRNA was not differentially expressed here. As the relative proportion of vessels and endothelial cells was much higher in the parietal samples than in the tumor samples one could expect that there was some overexpression of angiogenetic genes in the normal tissue due to a mass effect (Table 3). On the contrary there were very few genes differentially expressed, of the 25 genes associated to angiogenesis, GO:0001525, two of these genes were downregulated, namely ANG (angiogenin) and the PLXDC1 (plexin-domain containing protein 1). One gene was overexpressed in tumour, the AGGF1, a recently discovered potent angiogenic [59]. VG5Q, the protein encoded by this gene was overexpressed in >75% of tumour cells, also the sarcomatoid component, as well as the endothelium of pathologic vessels (Fig. 4 A–B). We have recently proposed this pro-angiogenic protein as a target for mesothelioma treatment[7]

### DNA repair and proteasome genes

DNA repair overexpression has recently been implicated in primary tumours with subsequent high metastatic potential, e.g. melanoma[42], and proteasome function interacts closely with some repair mechanisms [60], [61](Table 5). These repair systems have not been related to mesothelioma previously, and their possible implications for the extreme chemo- and radio-resistance of mesothelioma is discussed further in our recent paper[7].

### Cytokine-cytokine receptor interaction

Malignant tumours are generally known to express factors that modulate their environment, e.g. growth and pro-angiogenic factors, but are generally not responsive to normal control mechanisms of the microenvironment. Interestingly cytokine-cytokine receptor interaction pathways were severely altered by down-regulation of 19/21 genes in KEGG PATHWAYS (not shown), 197 genes of signal transduction and 33 out of 271 inflammatory genes (Table 6). The downregulated immune related genes belonged to the family of chemoattractants i.e, chemokines or growth factors i.e cytokines. Among those were several interleukin receptors and ligands (IL15, IL11RA, IL3RA and CSF2RB), the TGF-*β*- family receptor TGFBR2, and chemokine ligands (CXC and CC subfamily, TNF, TFSF14/LIGHT and BMP2). These are involved in inflammatory responses, chemotaxis of monocytes, activation of natural killer cells, but also in cancer suppression. Anomaly of these functions may be important for tumour progression. Loss of the tumour suppressor TGFBR2 expression is seen in many cancers with microsatellite instability and deleted in large-cell lung carcinoma[62]. Interestingly, array analysis showed that estrogen suppresses TGFBR2 gene in estrogen sensitive tumours[63], that could indicate a role of estrogen in mesothelioma as well. Leukocyte transendothelial migration genes were also downregulated, as discussed in our recent paper[7]. Only two immune genes were upregulated in the mesothelioma, one from the TGF-β family INHBE and one from the IL-1 receptor family the IL-1 receptor antagonistic peptide (IL1RAP). Interpretation of these findings could be that the tumour, the stroma or both are less permissive to cytokine activation and tumour suppressor activity due to down-regulation of cytokine receptor and ligands, a genotype with defect cell-cell communication facilitating progression and aggressive phenotype. The results also suggest that mesotheliomas effectively shut down attraction and activation of immune cells as an immune evasive mechanism.

### Susceptibility gene

Finally, mutation and dysfunction of the detoxifier GSTM1 is related to high risk of head and neck and lung cancer in smokers[64], [65]. Down-regulation of GSTM1 is a novel finding in mesothelioma, and its role in mesothelioma susceptibility should be evaluated.

### Study design and relevance of the samples

The study design as a whole was developed to avoid caveats of microarray analysis of complex tissues. Since initiaton and progression to a clinically detected malignant mesothelioma takes 20–60 years there are several unknown steps. We believe that our included control patients (relatively young, healthy and not exposed to asbestos) facilitated a true differential expression between malignant and healthy tissue. Lack of appropriate control samples in earlier studies may have been one reason for incongruent results[66], [67], [68], [69], [70], [71], [72], [73]. In spite of few cases and controls, the differential gene expression detected was highly significant.

The list of differentially expressed genes are based on a test that the average expression level is up or downregulated, however for as many as 519 of the reported upregulated genes and 542 of the downregulated there is no overlap in gene expression levels between the tumour and reference material. A large number of the genes that are found as differentially expressed represent pathways and biological processes widely known differentially regulated in cancer. The procedure to identify the genes takes both magnitude of change (fold change) and variability within the groups into account, and the p - values are corrected for multiple testing making it very likely that the reported genes are representative of changes even if the number of samples is low.

Some overexpressed genes were confirmed by immunohistochemistry and genes encoding proteins overexpressed in mesothelioma were also overexpressed here (e.g. Ki67, Syndecan 1, Survivin and Vitronectin). The genes FUT4 and ST6GALNAC3 coding for CD15 and Sialyl Transferase that are negative markers of mesothelioma, were down-regulated[74]. Unexpectedly the genes encoding the positive markers Calretinin, VEGFR and Mesothelin were not differentially expressed. However, recent studies showed that these are also expressed in normal mesothelial cells[75], [76], [77].

### Biopsies versus microdissected cells and cell lines

Mesothelioma arises in the pleura, but from which cell type? The mesothelial cell has been taken for granted as the progeny of mesothelioma, but recent studies showed that stem cells derived from adipose tissue, circulating multipotent fibrocytes and adult bone marrow-derived stem cells are able to transform to both epithelial and mesenchymal cells[78], [79], [80]. Thus, the progenitor cell could as well be a submesothelial fibrocyte/fibroblast or another stem cell type. Epithelial mesothelioma can transform to sarcomatoid phenotype[73], so one cannot argue that mesothelial cells become epithelial mesothelioma and that the sarcomatous type originate from fibrocytes/fibroblasts. Moreover, tumour stroma gene expression may differ from normal stroma[81], and its importance in tumour progression have recently been acknowledged. As a systems biology approach, profiling of tumour/stroma versus normal tissue/stroma may thus give important information on the interplay between cells in the microenvironment that would never be detected if only microdissected cells or cell lines were examined. Cultured cells also have the drawback of expressing other genes than malignant cells in situ, even changing expression according to number of passages[82] that further complicate the comparison.

Documentation of cell types and relative amount of each type by visual inspection of two-dimensional slides of adjacent tissue as done here was feasible and easy, but utmost important as the variability of cell content was high. For this reason we suggest that by any technique used to obtain material for comparisons of DNA or RNA from complex tissues, an evaluation of cell-types are should be pursued. This is, to our knowledge, the first mesothelioma microarray study to report the clinical status, histological description of cell types and an estimate of the proportion of cell types in biopsies from cases and controls. Even with a small number of samples with high variability in cell content we could see a differential expression of the three complex systems of cells, the tumour, the parietal and the visceral pleura.

### Conclusion

In conclusion, we have demonstrated a significant differential gene expression of mesothelioma, visceral and parietal pleura by genome-wide profiling, based on tissue samples that contained all the cell types normally seen. The highly malignant, resistant but non-metastatic phenotype of pleural mesothelioma was reflected in the present gene profile. Significant dysregulation of circadian rhythm genes may be important in driving the malignant process. An introvert and immunologically defensive genotype of mesothelioma was reflected by down-regulation of adhesion, cytokine receptors, ligands and inflammatory response genes. Normal parietal pleura showed downregulation of adhesion, solute transporter and signal tranduction systems that could confer to its susceptibility of transformation by asbestos. The results underscore the vast complexity of mesothelioma biology and that large-scale methods are necessary to reveal new functional pathological aspects, finally aiming at target discovery.

## Methods

### Ethics statement

The study protocol was approved by the Regional Committee of Research Ethics of Central Norway, the Health Departement and the Norwegian Social Science Data Service. Informed consent was obtained from all participants.

### Mesothelioma and control patients

Mesothelioma patients diagnosed between 2003–2005 were included. They were all subjected to a clinical examination and answered a patient history questionnaire. Diagnostic biopsies and material for gene expression were taken from adjacent locations with needle by Computer Tomography and/or ultrasound guidance. Diagnostic samples were formalin-fixed and paraffin-embedded. Material for gene expression analysis was snap-frozen in liquid nitrogen within two minutes. Biopsies of morphologically normal pleura were obtained from persons who underwent Video-Assisted Thoracoscopy (VATS) for recurrent pneumothorax, after obtaining patient history and informed consent. Parietal pleura that was stripped from the thoracic wall and visceral pleura dissected from the wedge-resections of the lung, were snap-frozen in liquid nitrogen within two minutes. Mesothelioma diagnosis was carried out by senior pathologists and re-examined by H. Sandeck, by including a standard panel of antibodies for immunohistochemistry as well as supplementary antibodies were used.

### Semi-quantitative histological description of adjacent tissue biopsies

Biopsies from tumour and control adjacent to the biopsies for microarray analysis, were examined histologically by H. Sandeck to identify which cell types were included in each specimen and also estimate the relative content of cells of each type (per cent of total cell nuclei).

### RNA-extraction

Methods used for RNA extraction were optimized to assure a high quality RNA from the small needle biopsies of the tumours. The final technique chosen was homogenization of frozen tissue with MagnaLyser (Roche Diagnostics) following the manufacturer's procedure 2×50 sec, but using 700 µL lysis buffer (Roche Diagnostics, Germany) as it gave higher RNA yield. The material was then incubated for 30 min at room temperature, centrifuged at 13000G for two minutes. 350 µL of the supernatant was used for further RNA isolation. Manual isolation with High Pure RNA Tissue Kit (Roche Diagnostics, Germany) according to the producer's protocol was performed. Quality control of RNA was done with NanoDrop (Saveen & Werner AB, Sweden) and Bioanalyzer (Agilent technologies, Inc. USA).

### Microarray experiments

Microarray experiments were performed at the Norwegian Microarray Consortium (NMC) at NTNU, Trondheim, Norway. Gene expression analysis was performed by the Affymetrix GeneChip system according to the manufacturer's Eukaryote Two-Cycle protocol, starting with 75 ng deep frozen total RNA. Labelled cRNA was hybridized to the Affymetrix Human Genome U133 Plus 2.0 GeneChip (Affymetrix, Santa Clara, CA, USA), of 38 500 genes and 47 000 trancripts, allowing genome-wide expression on a single array. The GeneChips were scanned using the GeneChip Scanner 3000 (Affymetrix). Quality controls were assessed using the GCOS v1.4 software, according to the manufacturer's manual (Affymetrix). All experiments have been submitted to ArrayExpress registered with accession number E-MTAB-47.

### Microarray statistical analysis

The raw probe set intensities were normalised by robust multi array average (RMA). Quality control was done of Benjamini and Hochberg[83], [84] and genes with corrected P-values smaller than 0.05 were taken as significant. The lists of significant genes were tested for overrepresentation in KEGG PATHWAYS (Kyoto Encyclopedia of Genes and Genomes)[53], and GO (gene ontology) terms[85] using Fishers exact test. The distribution of the gene expression pattern in significant pathways was visualised in the loading space of a bridge-partial least squares regression (PLS) model[86].

### Validation

Cell specific expression of proteins encoded by six selected genes, were validated by immunohistochemistry (respective gene symbols in brackets). The following antibodies were tested on fixed tissues adjacent to samples subjected to microarray. Thymidylate Synthase (TYMS ) (Millipore, USA) dilution 1∶50, VG5Q (AGGF1) (Abcam, Cambridge UK) dilution 1∶500, Chk1(CHEK1)(Epitomics, California, USA), dilution 1∶10, overnight incubation at −4°C, NQO1 (NQO1) (Zymed Laboratories, Carlsbad, CA, USA) dilution 1∶50, RAD21 (RAD21) (Abcam, Cambridge UK) dilution 1∶500 and mesothelin (MSLN)(Novocastra Laboratories, Newcastle, UK) dilution 1∶10, overnight incubation at −4°C. Selected positive and negative controls were included for all antibodies.

## References

[1] Robinson BW, Musk AW, Lake RA (2005). Malignant mesothelioma.. Lancet.

[2] Baas P (2005). Chemotherapy for malignant mesothelioma.. Lung Cancer.

[3] Bridda A, Padoan I, Mencarelli R, Frego M (2007). Peritoneal mesothelioma: a review.. MedGenMed.

[4] Boutin C, Rey F (1993). Thoracoscopy in pleural malignant mesothelioma: a prospective study of 188 consecutive patients. Part 1: Diagnosis.. Cancer.

[5] Lindholm PM, Salmenkivi K, Vauhkonen H, Nicholson AG, Anttila S (2007). Gene copy number analysis in malignant pleural mesothelioma using oligonucleotide array CGH.. Cytogenet Genome Res.

[6] Musti M, Kettunen E, Dragonieri S, Lindholm P, Cavone D (2006). Cytogenetic and molecular genetic changes in malignant mesothelioma.. Cancer Genet Cytogenet.

[7] Roe OD, Anderssen E, Sandeck H, Christensen T, Larsson E (2009). Malignant pleural mesothelioma: Genome-wide expression patterns reflecting general resistance mechanisms and a proposal of novel targets.. Lung Cancer.

[8] Roe OD, Creaney J, Lundgren S, Larsson E, Sandeck H (2008). Mesothelin-related predictive and prognostic factors in malignant mesothelioma: A nested case-control study.. Lung Cancer.

[9] Malard V, Berenguer F, Prat O, Ruat S, Steinmetz G (2007). Global gene expression profiling in human lung cells exposed to cobalt.. BMC Genomics.

[10] Wang NS (1985). Anatomy and physiology of the pleural space.. Clin Chest Med.

[11] Steiglitz BM, Keene DR, Greenspan DS (2002). PCOLCE2 encodes a functional procollagen C-proteinase enhancer (PCPE2) that is a collagen-binding protein differing in distribution of expression and post-translational modification from the previously described PCPE1.. J Biol Chem.

[12] Langsenlehner U, Renner W, Yazdani-Biuki B, Eder T, Wascher TC (2006). Integrin alpha-2 and beta-3 gene polymorphisms and breast cancer risk.. Breast Cancer Res Treat.

[13] FitzGerald LM, Patterson B, Thomson R, Polanowski A, Quinn S (2009). Identification of a prostate cancer susceptibility gene on chromosome 5p13q12 associated with risk of both familial and sporadic disease.. Eur J Hum Genet.

[14] Rustum YM, Takita H, Gomez G (1980). The design of cancer chemotherapy: metabolic modulation and cellular de novo versus salvage metabolism.. Antibiot Chemother.

[15] Kinsella AR, Haran MS (1991). Decreasing sensitivity to cytotoxic agents parallels increasing tumorigenicity in human fibroblasts.. Cancer Res.

[16] Rahman L, Voeller D, Rahman M, Lipkowitz S, Allegra C (2004). Thymidylate synthase as an oncogene: a novel role for an essential DNA synthesis enzyme.. Cancer Cell.

[17] de Angelis PM, Fjell B, Kravik KL, Haug T, Tunheim SH (2004). Molecular characterizations of derivatives of HCT116 colorectal cancer cells that are resistant to the chemotherapeutic agent 5-fluorouracil.. Int J Oncol.

[18] Mazurek S, Grimm H, Boschek CB, Vaupel P, Eigenbrodt E (2002). Pyruvate kinase type M2: a crossroad in the tumor metabolome.. Br J Nutr.

[19] Kumar Y, Tapuria N, Kirmani N, Davidson BR (2007). Tumour M2-pyruvate kinase: a gastrointestinal cancer marker.. Eur J Gastroenterol Hepatol.

[20] Hartsough MT, Clare SE, Mair M, Elkahloun AG, Sgroi D (2001). Elevation of breast carcinoma Nm23-H1 metastasis suppressor gene expression and reduced motility by DNA methylation inhibition.. Cancer Res.

[21] Gonzalez S, Klatt P, Delgado S, Conde E, Lopez-Rios F (2006). Oncogenic activity of Cdc6 through repression of the INK4/ARF locus.. Nature.

[22] Reimer D, Sadr S, Wiedemair A, Goebel G, Concin N (2006). Expression of the E2F family of transcription factors and its clinical relevance in ovarian cancer.. Ann N Y Acad Sci.

[23] Giacinti C, Giordano A (2006). RB and cell cycle progression.. Oncogene.

[24] Kamrul HM, Wadhwa R, Kaul SC (2007). CARF binds to three members (ARF, p53, and HDM2) of the p53 tumor-suppressor pathway.. Ann N Y Acad Sci.

[25] Hashimoto K, Araki K, Osaki M, Nakamura H, Tomita K (2004). MCM2 and Ki-67 expression in human lung adenocarcinoma: prognostic implications.. Pathobiology.

[26] Soling A, Sackewitz M, Volkmar M, Schaarschmidt D, Jacob R (2005). Minichromosome maintenance protein 3 elicits a cancer-restricted immune response in patients with brain malignancies and is a strong independent predictor of survival in patients with anaplastic astrocytoma.. Clin Cancer Res.

[27] Xu J, Zhang S, You C, Huang S, Cai B (2007). Expression of human MCM6 and DNA Topo II alpha in craniopharyngiomas and its correlation with recurrence of the tumor.. J Neurooncol.

[28] Ha SA, Shin SM, Namkoong H, Lee H, Cho GW (2004). Cancer-associated expression of minichromosome maintenance 3 gene in several human cancers and its involvement in tumorigenesis.. Clin Cancer Res.

[29] Winnepenninckx V, Lazar V, Michiels S, Dessen P, Stas M (2006). Gene expression profiling of primary cutaneous melanoma and clinical outcome.. J Natl Cancer Inst.

[30] Salabat MR, Melstrom LG, Strouch MJ, Ding XZ, Milam BM (2008). Geminin is overexpressed in human pancreatic cancer and downregulated by the bioflavanoid apigenin in pancreatic cancer cell lines.. Mol Carcinog.

[31] Acevedo-Duncan M, Patel R, Whelan S, Bicaku E (2002). Human glioma PKC-iota and PKC-betaII phosphorylate cyclin-dependent kinase activating kinase during the cell cycle.. Cell Prolif.

[32] Regala RP, Weems C, Jamieson L, Khoor A, Edell ES (2005). Atypical protein kinase C iota is an oncogene in human non-small cell lung cancer.. Cancer Res.

[33] Xu L, Deng X (2006). Suppression of cancer cell migration and invasion by protein phosphatase 2A through dephosphorylation of mu- and m-calpains.. J Biol Chem.

[34] Larochelle S, Merrick KA, Terret ME, Wohlbold L, Barboza NM (2007). Requirements for Cdk7 in the assembly of Cdk1/cyclin B and activation of Cdk2 revealed by chemical genetics in human cells.. Mol Cell.

[35] Castedo M, Perfettini JL, Roumier T, Kroemer G (2002). Cyclin-dependent kinase-1: linking apoptosis to cell cycle and mitotic catastrophe.. Cell Death Differ.

[36] Yu J, Guo QL, You QD, Zhao L, Gu HY (2007). Gambogic acid-induced G2/M phase cell-cycle arrest via disturbing CDK7-mediated phosphorylation of CDC2/p34 in human gastric carcinoma BGC-823 cells.. Carcinogenesis.

[37] Vivo C, Levy F, Pilatte Y, Fleury-Feith J, Chretien P (2001). Control of cell cycle progression in human mesothelioma cells treated with gamma interferon.. Oncogene.

[38] Turnell AS, Stewart GS, Grand RJ, Rookes SM, Martin A (2005). The APC/C and CBP/p300 cooperate to regulate transcription and cell-cycle progression.. Nature.

[39] Camasses A, Bogdanova A, Shevchenko A, Zachariae W (2003). The CCT chaperonin promotes activation of the anaphase-promoting complex through the generation of functional Cdc20.. Mol Cell.

[40] Prasanth SG, Prasanth KV, Stillman B (2002). Orc6 involved in DNA replication, chromosome segregation, and cytokinesis.. Science.

[41] Warner SL, Gray PJ, Von Hoff DD (2006). Tubulin-associated drug targets: Aurora kinases, Polo-like kinases, and others.. Semin Oncol.

[42] Kauffmann A, Rosselli F, Lazar V, Winnepenninckx V, Mansuet-Lupo A (2008). High expression of DNA repair pathways is associated with metastasis in melanoma patients.. Oncogene.

[43] Gery S, Koeffler HP (2007). The role of circadian regulation in cancer.. Cold Spring Harb Symp Quant Biol.

[44] Gery S, Komatsu N, Kawamata N, Miller CW, Desmond J (2007). Epigenetic silencing of the candidate tumor suppressor gene Per1 in non-small cell lung cancer.. Clin Cancer Res.

[45] Chen ST, Choo KB, Hou MF, Yeh KT, Kuo SJ (2005). Deregulated expression of the PER1, PER2 and PER3 genes in breast cancers.. Carcinogenesis.

[46] Xiang S, Coffelt SB, Mao L, Yuan L, Cheng Q (2008). Period-2: a tumor suppressor gene in breast cancer.. J Circadian Rhythms.

[47] Teboul M, Guillaumond F, Grechez-Cassiau A, Delaunay F (2008). The nuclear hormone receptor family round the clock.. Mol Endocrinol.

[48] Bertolucci C, Cavallari N, Colognesi I, Aguzzi J, Chen Z (2008). Evidence for an overlapping role of CLOCK and NPAS2 transcription factors in liver circadian oscillators.. Mol Cell Biol.

[49] Wood PA, Du-Quiton J, You S, Hrushesky WJ (2006). Circadian clock coordinates cancer cell cycle progression, thymidylate synthase, and 5-fluorouracil therapeutic index.. Mol Cancer Ther.

[50] Gorbacheva VY, Kondratov RV, Zhang R, Cherukuri S, Gudkov AV (2005). Circadian sensitivity to the chemotherapeutic agent cyclophosphamide depends on the functional status of the CLOCK/BMAL1 transactivation complex.. Proc Natl Acad Sci U S A.

[51] Tozlu-Kara S, Roux V, Andrieu C, Vendrell J, Vacher S (2007). Oligonucleotide microarray analysis of estrogen receptor alpha-positive postmenopausal breast carcinomas: identification of HRPAP20 and TIMELESS as outstanding candidate markers to predict the response to tamoxifen.. J Mol Endocrinol.

[52] Towne JE, Garka KE, Renshaw BR, Virca GD, Sims JE (2004). Interleukin (IL)-1F6, IL-1F8, and IL-1F9 signal through IL-1Rrp2 and IL-1RAcP to activate the pathway leading to NF-kappaB and MAPKs.. J Biol Chem.

[53] Kanehisa M, Araki M, Goto S, Hattori M, Hirakawa M (2008). KEGG for linking genomes to life and the environment.. Nucleic Acids Res.

[54] Briassouli P, Chan F, Savage K, Reis-Filho JS, Linardopoulos S (2007). Aurora-A regulation of nuclear factor-kappaB signaling by phosphorylation of IkappaBalpha.. Cancer Res.

[55] Lopez-Rios F, Chuai S, Flores R, Shimizu S, Ohno T (2006). Global gene expression profiling of pleural mesotheliomas: overexpression of aurora kinases and P16/CDKN2A deletion as prognostic factors and critical evaluation of microarray-based prognostic prediction.. Cancer Res.

[56] Mountzios G, Terpos E, Dimopoulos MA (2008). Aurora kinases as targets for cancer therapy.. Cancer Treat Rev.

[57] Ozdemir F, Akdogan R, Aydin F, Reis A, Kavgaci H (2006). The effects of VEGF and VEGFR-2 on survival in patients with gastric cancer.. J Exp Clin Cancer Res.

[58] Ohta Y, Shridhar V, Bright RK, Kalemkerian GP, Du W (1999). VEGF and VEGF type C play an important role in angiogenesis and lymphangiogenesis in human malignant mesothelioma tumours.. Br J Cancer.

[59] Tian XL, Kadaba R, You SA, Liu M, Timur AA (2004). Identification of an angiogenic factor that when mutated causes susceptibility to Klippel-Trenaunay syndrome.. Nature.

[60] Jacquemont C, Taniguchi T (2007). Proteasome function is required for DNA damage response and fanconi anemia pathway activation.. Cancer Res.

[61] Ogiso Y, Tomida A, Lei S, Omura S, Tsuruo T (2000). Proteasome inhibition circumvents solid tumor resistance to topoisomerase II-directed drugs.. Cancer Res.

[62] Wang JC, Su CC, Xu JB, Chen LZ, Hu XH (2007). Novel microdeletion in the transforming growth factor beta type II receptor gene is associated with giant and large cell variants of nonsmall cell lung carcinoma.. Genes Chromosomes Cancer.

[63] Wei T, Geiser AG, Qian HR, Su C, Helvering LM (2007). DNA microarray data integration by ortholog gene analysis reveals potential molecular mechanisms of estrogen-dependent growth of human uterine fibroids.. BMC Womens Health.

[64] Belogubova EV, Togo AV, Karpova MB, Kuligina E, Buslova KG (2004). A novel approach for assessment of cancer predisposing roles of GSTM1 and GSTT1 genes: use of putatively cancer resistant elderly tumor-free smokers as the referents.. Lung Cancer.

[65] Singh M, Shah PP, Singh AP, Ruwali M, Mathur N (2008). Association of genetic polymorphisms in glutathione S-transferases and susceptibility to head and neck cancer.. Mutat Res.

[66] Rihn BH, Mohr S, McDowell SA, Binet S, Loubinoux J (2000). Differential gene expression in mesothelioma.. FEBS Lett.

[67] Kettunen E, Nissen AM, Ollikainen T, Taavitsainen M, Tapper J (2001). Gene expression profiling of malignant mesothelioma cell lines: cDNA array study.. Int J Cancer.

[68] Singhal S, Wiewrodt R, Malden LD, Amin KM, Matzie K (2003). Gene expression profiling of malignant mesothelioma.. Clin Cancer Res.

[69] Mohr S, Keith G, Galateau-Salle F, Icard P, Rihn BH (2004). Cell protection, resistance and invasiveness of two malignant mesotheliomas as assessed by 10K-microarray.. Biochim Biophys Acta.

[70] Mohr S, Bottin MC, Lannes B, Neuville A, Bellocq JP (2004). Microdissection, mRNA amplification and microarray: a study of pleural mesothelial and malignant mesothelioma cells.. Biochimie.

[71] Hoang CD, D'Cunha J, Kratzke MG, Casmey CE, Frizelle SP (2004). Gene expression profiling identifies matriptase overexpression in malignant mesothelioma.. Chest.

[72] Kettunen E, Nicholson AG, Nagy B, Wikman H, Seppanen JK (2005). L1CAM, INP10, P-cadherin, tPA and ITGB4 over-expression in malignant pleural mesotheliomas revealed by combined use of cDNA and tissue microarray.. Carcinogenesis.

[73] Sun X, Wei L, Liden J, Hui G, Dahlman-Wright K (2005). Molecular characterization of tumour heterogeneity and malignant mesothelioma cell differentiation by gene profiling.. J Pathol.

[74] Dejmek A, Brockstedt U, Hjerpe A (1997). Optimization of a battery using nine immunocytochemical variables for distinguishing between epithelial mesothelioma and adenocarcinoma.. APMIS.

[75] Hassan R, Ho M (2008). Mesothelin targeted cancer immunotherapy.. Eur J Cancer.

[76] Lugli A, Forster Y, Haas P, Nocito A, Bucher C (2003). Calretinin expression in human normal and neoplastic tissues: a tissue microarray analysis on 5233 tissue samples.. Hum Pathol.

[77] Thickett DR, Armstrong L, Millar AB (1999). Vascular endothelial growth factor (VEGF) in inflammatory and malignant pleural effusions.. Thorax.

[78] Krause DS, Theise ND, Collector MI, Henegariu O, Hwang S (2001). Multi-organ, multi-lineage engraftment by a single bone marrow-derived stem cell.. Cell.

[79] Lama VN, Phan SH (2006). The extrapulmonary origin of fibroblasts: stem/progenitor cells and beyond.. Proc Am Thorac Soc.

[80] Boquest AC, Shahdadfar A, Brinchmann JE, Collas P (2006). Isolation of stromal stem cells from human adipose tissue.. Methods Mol Biol.

[81] Lu C, Bonome T, Li Y, Kamat AA, Han LY (2007). Gene alterations identified by expression profiling in tumor-associated endothelial cells from invasive ovarian carcinoma.. Cancer Res.

[82] Zanazzi C, Hersmus R, Veltman IM, Gillis AJ, van Drunen E (2007). Gene expression profiling and gene copy-number changes in malignant mesothelioma cell lines.. Genes Chromosomes Cancer.

[83] Reiner A, Yekutieli D, Benjamini Y (2003). Identifying differentially expressed genes using false discovery rate controlling procedures.. Bioinformatics.

[84] Benjamini Y, Hochberg Y (1995). Controlling the False Discovery Rate: A Practical and Powerful Approach to Multiple Testing.. Journal of the Royal Statistical Society.

[85] Ashburner M, Ball CA, Blake JA, Botstein D, Butler H (2000). Gene ontology: tool for the unification of biology. The Gene Ontology Consortium.. Nat Genet.

[86] Gidskehaug L, Anderssen E, Flatberg A, Alsberg BK (2007). A framework for significance analysis of gene expression data using dimension reduction methods.. BMC Bioinformatics.

